# Case report: Spindle cell sarcoma and pituitary adenoma in the sella region—a rare collision tumor

**DOI:** 10.3389/fonc.2024.1355119

**Published:** 2024-11-20

**Authors:** Peng-fei Ding, Ting Zhu, Yue Cui, Hui-ying Yan, Yun-feng Wang, Chun-hua Hang, Wei Li

**Affiliations:** ^1^ Department of Neurosurgery, Nanjing Drum Tower Hospital Clinical College of Nanjing Medical University, Nanjing, China; ^2^ Department of Neurosurgery, Nanjing Drum Tower Hospital, Affiliated Hospital of Medical School, Nanjing University, Nanjing, China; ^3^ Department of Neurosurgery, Nanjing Drum Tower Hospital Clinical College of Nanjing University of Chinese Medicine, Nanjing, China

**Keywords:** collision tumor, sella region, spindle cell sarcoma, pituitary adenoma, case report

## Abstract

**Background:**

Collision tumors are defined as two or more distinctly bordered, mutually invasive tumors in the same anatomical region. Characterized by low incidence and lack of specificity, they often pose a significant challenge to disease diagnosis. Among these, collision tumors in the sella region are incredibly rare.

**Case description:**

On 13 June 2023, the Department of Neurosurgery at Drum Tower Hospital admitted a patient with a pituitary adenoma in the sella region complicated by spindle cell sarcoma. After reviewing the literature, no related cases were reported. A patient presenting with complex clinical symptoms and preoperative imaging showing occupancy in the sella region is considered to have a recurrence of pituitary adenoma. After thorough preoperative evaluation and discussion, a trans-sphenoidal approach (with the aid of an endoscope) was performed to resect the pituitary lesions. Combined with postoperative pathological tissue and imaging, the patient was diagnosed with a pituitary adenoma (postoperative recurrence) in the sella region complicated by spindle cell sarcoma. The patient made a fair recovery and was discharged on the 8th postoperative day.

**Conclusion:**

This case report aims to elucidate and discuss the diagnosis and screening of rare collision tumors in the sella region to reduce the misdiagnosis rate and provide accurate treatment.

## 1 Introduction

A pituitary adenoma is a common tumor in the sella region, while spindle cell sarcoma is rarely reported in there (1, 2). Only one case of spindle cell sarcoma in the sella region has been reported in previous studies (3). Two or more clearly bounded, mutually invasive tumors in the same anatomical location are referred to as collision tumors. Collision tumors in the sella region are extremely rare, and the co-existence of a pituitary adenoma with spindle cell sarcoma has never been reported. To better summarize the diagnosis and treatment of sella collision tumors, we first performed a literature review of collision tumors in the sella region after describing this case. We then excluded non-English articles, cases involving Rathke’s cyst, and those with ambiguous classifications of pituitary adenomas, and finally categorized the remaining studies according to the biological composition of the collision tumors (**Supplementary Table S1**).

## 2 Case description

**Figure 1:**
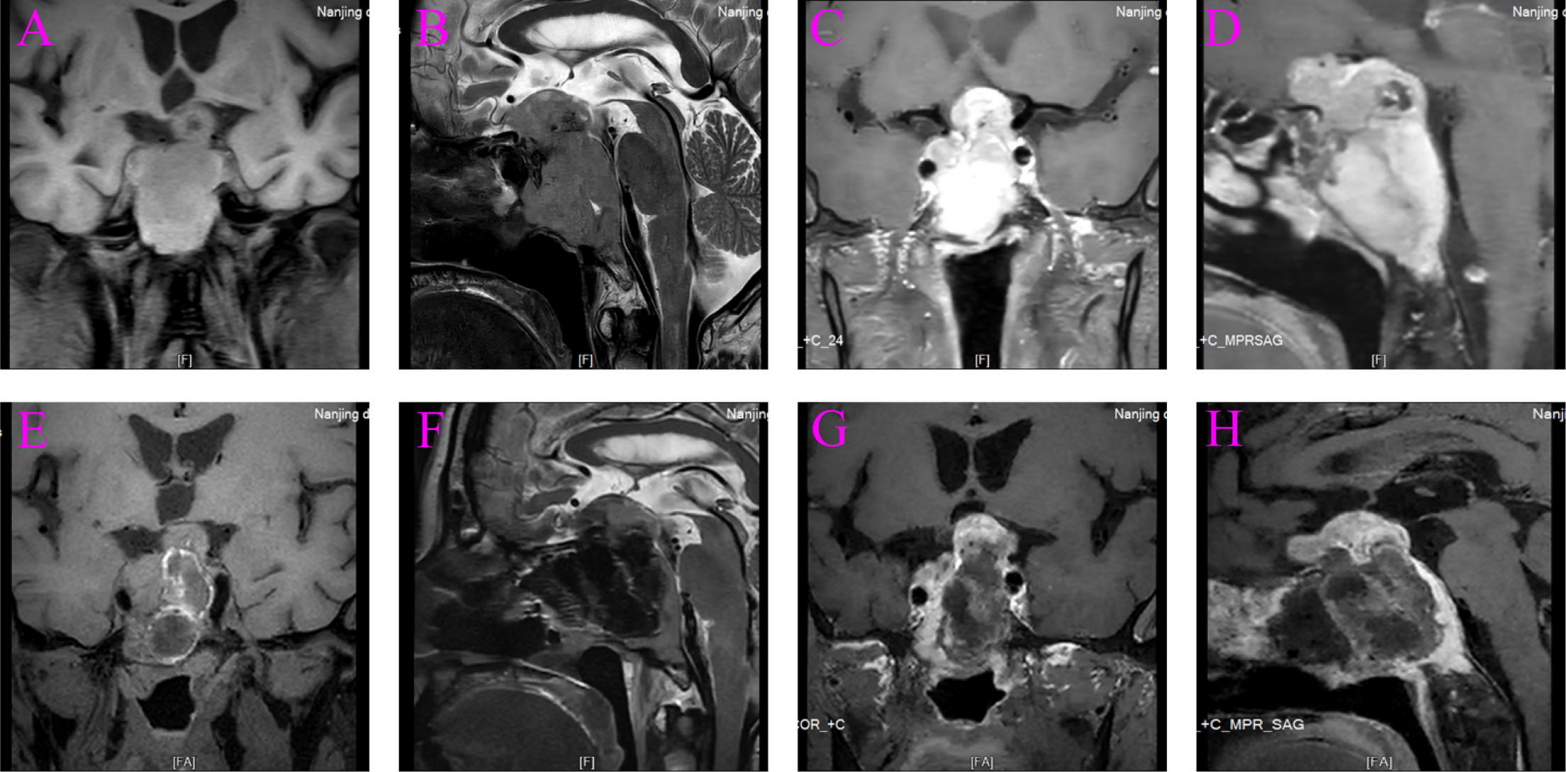
Preoperative and postoperative magnetic resonance imaging (MRI). **(A)** Preoperative coronal T1-weighted MRI. **(B)** Preoperative sagittal T2-weighted MRI. **(C)** Preoperative coronal T1-weighted with contrast. **(D)** Preoperative sagittal T1-weighted with contrast. **(E)** Postoperative coronal T1-weighted MRI. **(F)** Postoperative sagittal T2-weighted MRI. **(G)** Postoperative coronal T1-weighted with contrast. **(H)** Postoperative sagittal T1-weighted with contrast.

**Figure 2:**
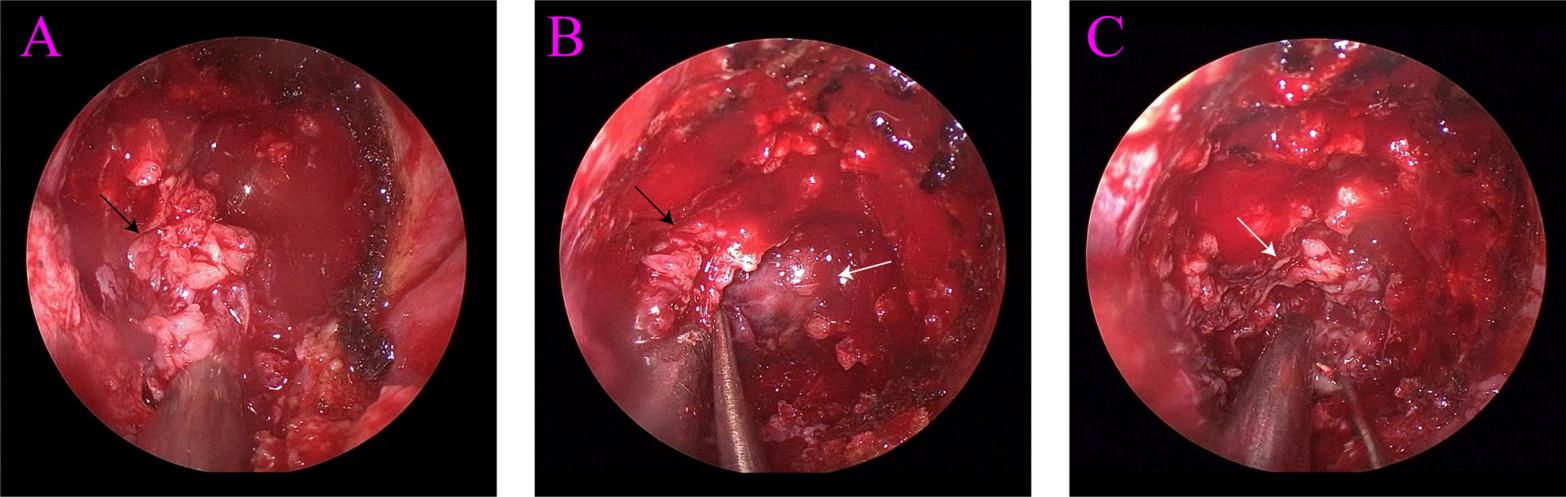
**(A)** Spindle cell sarcoma (black arrow). **(B)** Spindle cell sarcoma (black arrow) and pituitary adenoma (white arrow). **(C)** Pituitary adenoma (white arrow).

**Figure 3:**
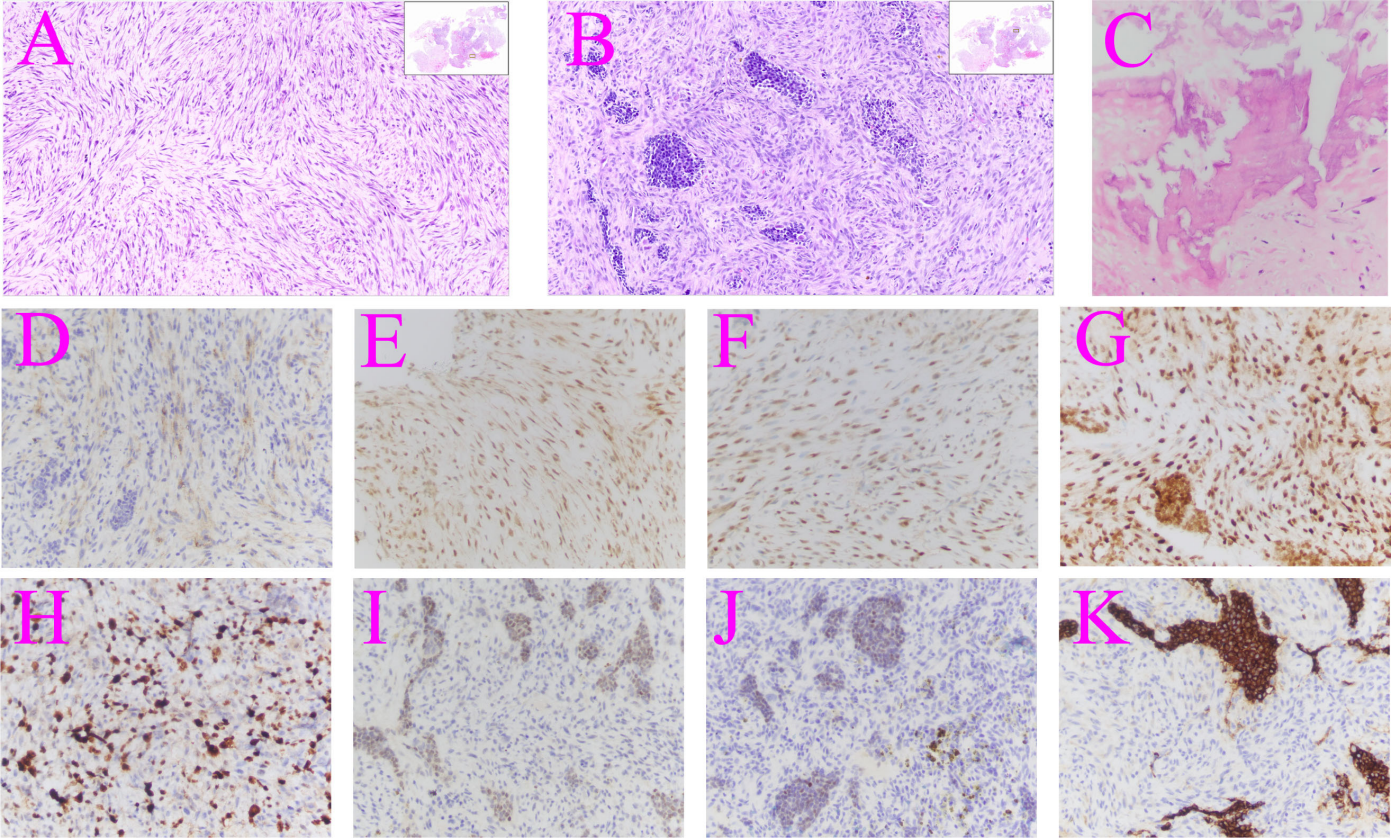
Hematoxylin–eosin and immunohistochemical staining of the surgically resected tumor. **(A)** Spindle cell sarcoma (×10). **(B)** Pituitary adenoma (×10). **(C)** Focal osteogenesis (×40). **(D)** EMA: focal positive immunohistochemistry (×40). **(E)** MDM2: positive immunohistochemistry (×40). **(F)** CDK4: positive immunohistochemistry (×40). **(G)** SATB2: positive immunohistochemistry (×40). **(H)** Ki-67 positive cells (×40). **(I)** SPF-1: positive immunohistochemistry (×40). **(J)** INSM1: positive immunohistochemistry (×40). **(K)** Synaptophysin: positive immunohistochemistry (×40).

A 67-year-old man was admitted to the hospital with complaints of “headache and fatigue for half a month, accompanied by nausea and vomiting several times”. The patient initially visited a local hospital, where a cranial MRI revealed a mass in the sella region. Considering that the patient had undergone a trans-sphenoidal pituitary adenoma resection in 2009, the local hospital diagnosed a recurrence of pituitary adenoma. The patient sought further treatment at Nanjing Drum Tower Hospital. Physical examination revealed no obvious abnormalities except for bilateral vision impairment and visual field deficits. Endocrine laboratory tests showed lower levels of triiodothyronine (T3), luteinizing hormone (LH), progesterone, and testosterone (**Table 1**). Preoperative cranial MRI revealed a mass in the sella region, characterized by an enlarged pituitary fossa and inhomogeneous abnormal signals. The larger mass measured approximately 1.5 cm × 1.0 cm, with the pituitary gland and pituitary stalk poorly visualized. The lesion exhibited inhomogeneous enhancement following contrast administration (**Figures 1A–D**). Based on the patient’s clinical manifestations, physical examination, and auxiliary tests, the main preliminary diagnosis was pituitary adenoma (postoperative recurrence). After the exclusion of contraindications to surgery, a trans-sphenoidal resection of the pituitary lesions was performed. During the operation, it was noted that the bone of the anterior wall of the sella region was partially absent. The tumor had a firm texture, a relatively rich grayish-white blood supply, and exhibited aggressive growth, extending into the cavernous sinuses, the slopes, and the anterior cranial base on both sides. Its size was approximately 5.0 cm × 4.0 cm × 3.5 cm (**Figure 2**). Endoscopic intratumoral decompression was performed, followed by gradual resection of the tumor. Postoperative pathological findings include the following: pituitary adenoma consistent with recurrent SF-1 spectrum, immunophenotype indicating gonadotropin cell tumor; sella spindle cell dysplasia predisposes to sella spindle cell sarcoma with focal ossification. Hematoxylin–eosin (HE) staining showed that the spindle cells were interwoven, exhibiting certain atypia and focal osteogenesis (**Figures 3A, C**). Additionally, small blue cells with round nuclei and rich eosinophilic cytoplasm were scattered in nests within the spindle cell lesions (**Figure 3B**). Immunohistochemical staining showed that the expression of EMA, MDM2, CDK4, and SATB2 in spindle cells was positive (**Figures 3D–G**), whereas GFAP, Desmin, CD34, S100, ALK, and TTF-1 were negative. The Ki-67 positive index was approximately 25% (**Figure 3H**). The expression of SF-1, INSM1, and Synaptophysin was positive in the scattered small blue cells (**Figures 3I–K**), while PIT-1, T-PIT, GH, LH, ACTH, PRL, FSH, TSH, and CK were negative. Molecular pathological results showed that TERT promoter mutation was negative (fluorescent PCR), BRAF V600E mutation was negative (fluorescent PCR), and CDKN2A homozygous deletion was negative (FISH). Postoperative head MRI revealed mixed signals with irregular enhancement in the sella region following pituitary adenoma surgery (**Figures 1E–H**). After the surgery, endocrine laboratory tests indicated that levels of T3, LH, progesterone, and testosterone remained below normal, necessitating further hormone therapy (**Table 1**). Following surgery, the headache symptoms and visual defect significantly improved, and no recurrence was observed after three months of follow-up.

**Table 1:** Comparative endocrinologic tests before and after surgery.

	Hormones			
	T3 (normal range: 3.1–6.8 pmol/L)	LH (normal range: 1.5–9.3 mIU/mL)	Progesterone (normal range: 0.89–3.88 nmol/L)	Testosterone (normal range: 6.51–3.23.74 nmol/L)
Preoperative	2.85	1.16	0.67	0.74
postoperative	2.74	0.9	0.67	0.28

## 3 Discussion

The synchronous occurrence of a pituitary adenoma and another tumor in the sella region is exceedingly rare, and the underlying mechanisms of this phenomenon remain unclear (4). In the case reports we summarized, visual deficits and headaches were the most common symptoms. Our patient similarly exhibited these symptoms (5–12). Misdiagnosis is a significant risk due to the nonspecific nature of the symptoms and imaging associated with collision tumors. The only way to determine the type of collision tumor is through histological analysis following tumor resection (13). Timely diagnosis and a clear understanding of the composition of collision tumors are very important for the prognosis of patients.

Sarcomas are widespread, heterogeneous, and biologically diverse tumors with the shared characteristic of mesenchymal cell origin (14). Historically, sarcomas have been classified into two major groups: soft tissue sarcomas and bone sarcomas (15). The immunohistochemical analysis of MDM2, CDK4, and SATB2 in this case report was positive, indicating that this spindle cell sarcoma belongs to the category of bone sarcomas. Although there is still no conclusive proof, postoperative radiotherapy for pituitary adenoma may be linked to the emergence of sarcomas (2, 16, 17). Spindle cell sarcoma in the sella region is a very rare occurrence. Sareen et al. reported a case of primary undifferentiated spindle cell sarcoma in the sella region without any history of radiation therapy (3). In this case report, the patient similarly did not receive radiation therapy after surgery for a pituitary adenoma many years earlier. The most effective way to treat sarcoma is through complete resection of the tumor. However, this is not always possible due to the limited potential space in the sella region. Radiotherapy is typically used in patients with persistent and recurrent sarcoma, while chemotherapy is less standardized because different subtypes of sarcoma have different chemosensitivity (2). The Ki-67 positive index for spindle cell sarcoma in this case is 25%. Given its relatively low level of proliferation, the tumor was considered less aggressive, so radiotherapy and chemotherapy were not administered following surgical removal.

As the reference provided classifies the pituitary adenomas based on hormonal activity into two main categories—nonfunctioning (or nonsecreting) and functioning (or secreting) pituitary adenomas—the latter includes prolactinomas, somatotropinomas, corticotropinomas, and thyrotropinomas (1). Except for thyrotropinomas, which make up a very minor part of pituitary adenomas, **Supplementary Table S1** revealed that the other four categories of pituitary adenomas have been reported to coexist with other tumor types in the sella region. Nonfunctional adenomas, which make up the second-most pituitary adenomas, are recorded as collision tumors more frequently than prolactinomas, which account for the majority of pituitary adenomas. Among these reports, the most frequent combination involves a nonfunctional adenoma and a craniopharyngioma. Although conclusive data are lacking, Bteich et al. hypothesized that the formation mechanism of collision tumors may be due to nonfunctional adenomas and craniopharyngiomas sharing the same embryonic origin—Rathke’s pouch (8). Additionally, Ban et al. suggested that pituitary hormones such as FSH, GH, PRL, and TSH can bind to hormone receptors present in lymphoid tissue, stimulate the growth of lymphocytes, and ultimately lead to the co-occurrence of pituitary adenoma and lymphoma (13).

In this case, we present a 67-year-old man suffering from a nonfunctioning pituitary adenoma complicated by spindle cell sarcoma, a combination that has never been reported before. The symptoms of the patient at admission were not significantly different from those of conventional pituitary adenoma, which led to an initial diagnosis at a local hospital of postoperative recurrence of adenoma. Preoperative head MRI revealed several uneven aberrant signals in the sella region, with uneven enhancement of the lesion observed on enhanced scan. Determining the exact characteristics of the tumors before surgery was challenging. During the procedure, two distinct tumors were identified with clear boundaries between them (**Figure 2B**). Surgery remains the only available treatment for a sella collision tumor. However, because the tumor frequently extends to the pituitary stalk, hypothalamus, third ventricle, cerebellar horn, and infratemporal region due to the tiny potential space of the sella, the surgery is extremely difficult (10). When a patient’s sella region MRI reveals abnormal heterogeneity, it is important to carefully review the scan with the imaging department. FDG PET/CT can be a valuable tool for distinguishing between pituitary adenoma and spindle cell sarcoma. Pituitary adenomas, generally benign and slow-growing, typically show variable FDG uptake, with mild to moderate intensity depending on their subtype and metabolic activity, and it has an SUVmax cutoff of 4.1. In contrast, spindle cell cancers, which are rare and aggressive malignancies, often demonstrate significantly higher FDG uptake due to their hypermetabolic nature. The intense FDG avidity seen in spindle cell cancers can help differentiate them from pituitary adenomas when MRI findings are inconclusive or when there is suspicion of malignancy (18, 19). An alternative surgical plan should be prepared for the patient who may have a collision tumor in the sella region. The tumor boundary should be precisely delineated and carefully eliminated during the procedure, and the presence of the second tumor should not be disregarded. To create the following treatment plan more accurately, the nature of the surgically removed tumor must be determined through histological studies.

## 4 Conclusions

Collision tumors in the sella region are extremely rare and difficult to diagnose clinically. Only a histological test can determine the presence of concurrent tumors, as CT and MRI cannot distinguish between two separate tumors. This is the first reported instance of a pituitary adenoma and spindle cell sarcoma coexisting in the sella region. Based on previous case reports and our expertise in diagnosing and treating sella collision tumors, we believe that prompt surgical removal of the tumor, followed by histological examination to ascertain the exact type of tumor coexisting with the pituitary adenoma, may be crucial for improving the patient’s prognosis.

## Data Availability

The original contributions presented in the study are included in the article/**Supplementary Material**. Further inquiries can be directed to the corresponding author.

## References

[1] Tritos NA Miller KK. Diagnosis and management of pituitary adenomas. Jama. (2023) 329:1386–98. doi: 10.1001/jama.2023.5444 37097352

[2] Guerrero-Pérez F Vidal N López-Vázquez M Sánchez-Barrera R Sánchez-Fernández JJ Torres-Díaz A. Sarcomas of the sellar region: a systematic review. Pituitary. (2020) 24:117–29. doi: 10.1007/s11102-020-01073-9 32785833

[3] Sareen P Chhabra L Trivedi N. Primary undifferentiated spindle-cell sarcoma of sella turcica: successful treatment with adjuvant temozolomide. Case Rep. (2013) 2013:bcr2013009934–bcr. doi: 10.1136/bcr-2013-009934 PMC3670048 23715844

[4] Lamorie-Foote K Rangwala SD Kammen A Gnass E Kramer DR Rutkowski M. Melanoma metastasis to a nonfunctioning pituitary macroadenoma: illustrative case. J Neurosurgery: Case Lessons. (2021) 1. doi: 10.3171/CASE2167 PMC9394700 36046510

[5] Jin G Hao S Xie J Mi R Liu F. Collision tumors of the sella: coexistence of pituitary adenoma and craniopharyngioma in the sellar region. World J Surg Oncol. (2013) 11:178. doi: 10.1186/1477-7819-11-178 23919255 PMC3750462

[6] Miyazaki T Kowari K Eda H Kambara M Maruyama R Akiyama Y. Ten-year follow-up of collision tumors composed of craniopharyngioma and pituitary adenoma: A case report and literature review. Case Rep Med. (2019) 2019:1–7. doi: 10.1155/2019/8080163 PMC6664541 31396283

[7] Kikuta H Jinguji S Sato T Bakhit M Hiruta R Sato Y. A collision tumor of pit-1/SF-1-positive double pituitary adenoma and a craniopharyngioma coexisting with graves' Disease. NMC Case Rep J. (2023) 10:169–75. doi: 10.2176/jns-nmc.2022-0396 PMC10310352 37398916

[8] Bteich F El Khoury L Nohra G Trak V Yazbek S Akiki M. Pituitary adenoma and papillary craniopharyngioma: A rare case of collision tumor and review of the literature. World Neurosurgery. (2020) 139:63–9. doi: 10.1016/j.wneu.2020.03.088 32298831

[9] Shakally A Tahara N Clark B Tummala R Caicedo-Granados E Kawakami Y. A rare case of recurrent pituitary collision tumors. J Endocrine Soc. (2020) 4. doi: 10.1210/jendso/bvaa089 PMC7412853 32783016

[10] Shareef Z Kerndt C Nessel T Mistry D Figueroa B. Collision tumor in the pituitary, concurrent pituitary adenoma, and craniopharyngioma. Case Rep Otolaryngology. (2020) 2020:1–5. doi: 10.1155/2020/9584090 PMC7495153 32963865

[11] Gokden M Mrak RE. Pituitary adenoma with craniopharyngioma component. Hum Pathology. (2009) 40:1189–93. doi: 10.1016/j.humpath.2009.02.007 19427020

[12] Moshkin O Scheithauer BW Syro LV Velasquez A Horvath E Kovacs K. Collision tumors of the sella: craniopharyngioma and silent pituitary adenoma subtype 3: case report. Endocrine Pathology. (2009) 20:50–5. doi: 10.1007/s12022-009-9065-3 19238590

[13] Ban VS Chaudhary BR Allinson K Santarius T Kirollos RW. Concomitant primary CNS lymphoma and FSH-pituitary adenoma arising within the sella. Entirely Coincidental? Neurosurgery. (2017) 80:E170–E5. doi: 10.1093/neuros/nyw003 PMC5808144 28362886

[14] Hui JY. Epidemiology and etiology of sarcomas. Surg Clin North Am. (2016) 96:901–14. doi: 10.1016/j.suc.2016.05.005 27542634

[15] Hatina J Kripnerova M Houfkova K Pesta M Kuncova J Sana J. Sarcoma stem cell heterogeneity. Adv Exp Med Biol. (2019) 1123:95–118. doi: 10.1007/978-3-030-11096-3_7 31016597

[16] Kurosaki M Kambe A Ishibashi M Watanabe T Horie Y. Case report of sarcoma of the sella caused by postoperative radiotherapy for a prolactin-producing pituitary adenoma. Brain Tumor Pathol. (2014) 31:187–91. doi: 10.1007/s10014-014-0175-3 24446079

[17] Yamanaka R Abe E Sato T Hayano A Takashima Y. Secondary intracranial tumors following radiotherapy for pituitary adenomas: A systematic review. Cancers (Basel). (2017) 9. doi: 10.3390/cancers9080103 PMC5575606 28786923

[18] Hyun SH Choi JY Lee KH Choe YS Kim BT. Incidental focal 18F-FDG uptake in the pituitary gland: clinical significance and differential diagnostic criteria. J Nucl Med. (2011) 52:547–50. doi: 10.2967/jnumed.110.083733 21421711

[19] Hirmas N Hamacher R Sraieb M Ingenwerth M Kessler L Pabst KM. Fibroblast-activation protein PET and histopathology in a single-center database of 324 patients and 21 tumor entities. J Nucl Med. (2023) 64:711–6. doi: 10.2967/jnumed.122.264689 36581374

