# Supplementary material for: Case report: Spindle cell sarcoma and pituitary adenoma in the sella region—a rare collision tumor
Source: Front Oncol. 2024 Nov 20;14:1355119. doi: 10.3389/fonc.2024.1355119 (PMC11614765; doi:10.3389/fonc.2024.1355119)
Supplement: Supplementary file 1 [file DataSheet1.docx]

Supplementary table 1．Cases of Coexisting Pituitary Adenoma and Other Sella Tumors that Have Been Reported

| **Type of pituitary adenomas** | **Type of other tumors** | **Reference** |
| --- | --- | --- |
| **Non-functioning pituitary adenoma** | **Craniopharyngioma** | **(1-8)** |
|  | **Anaplastic astrocytoma** | **(9)** |
|  | **Chondroma** | **(10)** |
|  | **Gangliocytoma** | **(11)** |
|  | **Lung Adenocarcinoma** | **(12)** |
|  | **Lymphoma** | **(13)** |
|  | **Melanoma** | **(14)** |
|  | **Metastatic lung cancer** | **(15)** |
|  | **Spindle cell sarcoma** | **Present study** |
| **Prolactinoma** | **Lymphoma** | **(16)** |
|  | **Plasmocytoma** | **(17)** |
| **Somatotropinoma** | **Gangliocytoma** | **(18, 19)** |
|  | **Gonadotroph** | **(20)** |
|  | **Meningioma** | **(21)** |
|  | **Gangliocytoma and ganglioglioma** | **(22)** |
| **Corticotropinoma** | **Craniopharyngioma** | **(23, 24)** |
|  | **Meningioma** | **(25)** |

1. Jin G, Hao S, Xie J, Mi R, Liu F. Collision tumors of the sella: coexistence of pituitary adenoma and craniopharyngioma in the sellar region. World J Surg Oncol. 2013;11:178.

2. Miyazaki T, Kowari K, Eda H, Kambara M, Maruyama R, Akiyama Y. Ten-Year Follow-Up of Collision Tumors Composed of Craniopharyngioma and Pituitary Adenoma: A Case Report and Literature Review. Case Reports in Medicine. 2019;2019:1-7.

3. Kikuta H, Jinguji S, Sato T, Bakhit M, Hiruta R, Sato Y, et al. A Collision Tumor of Pit-1/SF-1-positive Double Pituitary Adenoma and a Craniopharyngioma Coexisting with Graves' Disease. NMC Case Report Journal. 2023;10(0):169-75.

4. Bteich F, El Khoury L, Nohra G, Trak V, Yazbek S, Akiki M. Pituitary Adenoma and Papillary Craniopharyngioma: A Rare Case of Collision Tumor and Review of the Literature. World Neurosurgery. 2020;139:63-9.

5. Shakally A, Tahara N, Clark B, Tummala R, Caicedo-Granados E, Kawakami Y, et al. A Rare Case of Recurrent Pituitary Collision Tumors. Journal of the Endocrine Society. 2020;4(9).

6. Shareef Z, Kerndt C, Nessel T, Mistry D, Figueroa B. Collision Tumor in the Pituitary, Concurrent Pituitary Adenoma, and Craniopharyngioma. Case Reports in Otolaryngology. 2020;2020:1-5.

7. Gokden M, Mrak RE. Pituitary adenoma with craniopharyngioma component. Human Pathology. 2009;40(8):1189-93.

8. Moshkin O, Scheithauer BW, Syro LV, Velasquez A, Horvath E, Kovacs K. Collision Tumors of the Sella: Craniopharyngioma and Silent Pituitary Adenoma Subtype 3: Case Report. Endocrine Pathology. 2009;20(1):50-5.

9. Naik H, Vernon V, Gade P, Bhople L, Guha A. Anaplastic astrocytoma and pituitary macroadenoma within the same patient: A rare case of intracranial collision tumor. Neurol India. 2018;66(3):857-60.

10. Sahli R, Christ E, Kuhlen D, Giger O, Vajtai I. Sellar collision tumor involving pituitary gonadotroph adenoma and chondroma: a potential clinical diagnosis. Pituitary. 2009;14(4):405-8.

11. Heng L-J, Jia D, Gong L, Zhang W, Ma J, Qu Y. Endoscopic Endonasal Resection of a Mixed Lesion of Gangliocytoma and Nonfunctioning Pituitary Adenoma. World Neurosurgery. 2017;106:1050.e1-.e6.

12. Borhan MK, Tan FHS, Basry NSA. Collision of Two Tumors: A Case Report of a Lung Adenocarcinoma With Metastasis to a Pituitary Adenoma. Journal of the ASEAN Federation of Endocrine Societies. 2022;37(2):89-94.

13. Ban VS, Chaudhary BR, Allinson K, Santarius T, Kirollos RW. Concomitant Primary CNS Lymphoma and FSH-Pituitary Adenoma Arising Within the Sella. Entirely Coincidental? Neurosurgery. 2017;80(1):E170-E5.

14. Lamorie-Foote K, Rangwala SD, Kammen A, Gnass E, Kramer DR, Rutkowski M, et al. Melanoma metastasis to a nonfunctioning pituitary macroadenoma: illustrative case. Journal of Neurosurgery: Case Lessons. 2021;1(23).

15. Sogani J, Yang W, Lavi E, Zimmerman RD, Gupta A. Sellar collision tumor involving metastatic lung cancer and pituitary adenoma: radiologic-pathologic correlation and review of the literature. Clinical Imaging. 2014;38(3):318-21.

16. Ren S, Lu Q, Xiao Y, Zhang Y, Zhang L, Li B, et al. Coexistence of Pituitary Adenoma and Primary Pituitary Lymphoma: A Case Report and Review of the Literature. Frontiers in Surgery. 2022;9.

17. Rivera J, Alves S, Bianchi CC, Al-Mutawa N, Guiot MC, Zeitouni A. An unusual collision tumor comprising a prolactinoma and a plasmocytoma originating from the sellar region. Pituitary. 2008;13(2):189-93.

18. Tanriover N, Aydin O, Kucukyuruk B, Abuzayed B, Guler H, Oz B, et al. Endoscopic Approach to a Collision Tumor of Growth Hormone-Secreting Adenoma and Gangliocytoma in the Pituitary Gland. Journal of Craniofacial Surgery. 2014;25(4):1277-9.

19. Levitus CF, Charitou MM. An Incidental Collision Tumor of the Sella Turcica. AACE Clinical Case Reports. 2019;5(4):e247-e9.

20. Syro LV, Horvath E, Kovacs K. Double adenoma of the pituitary: a somatotroph adenoma colliding with a gonadotroph adenoma. J Endocrinol Invest. 2000;23(1):37-41.

21. Zhao Y, Zhang H, Lian W, Xing B, Feng M, Liu X, et al. Collision tumors composed of meningioma and growth hormone-secreting pituitary adenoma in the sellar region. Medicine. 2017;96(50).

22. Jukes A, Allan R, Rawson R, Buckland ME. Growth hormone secreting pituitary adenoma with admixed gangliocytoma and ganglioglioma. Journal of Clinical Neuroscience. 2016;31:202-4.

23. Finzi G, Cerati M, Marando A, Zoia C, Ferreli F, Tomei G, et al. Mixed pituitary adenoma/craniopharyngioma: clinical, morphological, immunohistochemical and ultrastructural study of a case, review of the literature, and pathogenetic and nosological considerations. Pituitary. 2013;17(1):53-9.

24. Snyder R, Fayed I, Dowlati E, Seager A, Mason RB. Pituitary Adenoma and Craniopharyngioma Collision Tumor: Diagnostic, Treatment Considerations, and Review of the Literature. World Neurosurgery. 2019;121:211-6.

25. Gezer E, Cantürk Z, Selek A, Çetinarslan B, Tarkun İ, Sözen M, et al. Cushing’s disease due to a pituitary adenoma as a component of collision tumor: A case report and review of the literature. Journal of Medical Case Reports. 2020;14(1).
